# Clinical and temporal patterns of severe pneumonia causing critical illness during Hajj

**DOI:** 10.1186/1471-2334-12-117

**Published:** 2012-05-16

**Authors:** Yasser Mandourah, Assim Al-Radi, Ali Harold Ocheltree, Sara Rashid Ocheltree, Robert A Fowler

**Affiliations:** 1 Department of Intensive Care, Riyadh Military Hospital, P.O. Box 7897, 11159, Riyadh, Kingdom of Saudi Arabia; 2 Department of Oncology, Oncology Center at King Abdul Aziz Hospital and Oncology Center, Jeddah, Kingdom of Saudi Arabia; 3 Department of Internal Medicine, North West Armed Forces Hospital, Tabuk, Kingdom of Saudi Arabia; 4 Department of Intensive Care, King Abdul-Aziz Hospital and Oncology Center, Jeddah, Kingdom of Saudi Arabia; 5 Department of Critical Care Medicine and Department of Medicine Sunnybrook Hospital, University of Toronto, Toronto, Canada

**Keywords:** Respiratory tract infection, Pneumonia, Hajj, Co–morbidities, APACHE IV

## Abstract

**Background:**

Pneumonia is a leading cause of hospitalization during Hajj and susceptibility and transmission may be exacerbated by extreme spatial and temporal crowding. We describe the number and temporal onset, co–morbidities, and outcomes of severe pneumonia causing critical illness among pilgrims.

**Method:**

A cohort study of all critically ill Hajj patients, of over 40 nationalities, admitted to 15 hospitals in 2 cities in 2009 and 2010. Demographic, clinical, and laboratory data, and variables necessary for calculation of the Acute Physiology and Chronic Health Evaluation IV scores were collected.

**Results:**

There were 452 patients (64.6% male) who developed critical illness. Pneumonia was the primary cause of critical illness in 123 (27.2%) of all intensive care unit (ICU) admissions during Hajj. Pneumonia was community (Hajj)–acquired in 66.7%, aspiration–related in 25.2%, nosocomial in 3.3%, and tuberculous in 4.9%. Pneumonia occurred most commonly in the second week of Hajj, 95 (77.2%) occurred between days 5–15 of Hajj, corresponding to the period of most extreme pilgrim density. Mechanical ventilation was performed in 69.1%. Median duration of ICU stay was 4 (interquartile range [IQR] 1–8) days and duration of ventilation 4 (IQR 3–6) days. Commonest preexisting co–morbidities included smoking (22.8%), diabetes (32.5%), and COPD (17.1%). Short–term mortality (during the 3–week period of Hajj) was 19.5%.

**Conclusion:**

Pneumonia is a major cause of critical illness during Hajj and occurs amidst substantial crowding and pilgrim density. Increased efforts at prevention for at risk pilgrim prior to Hajj and further attention to spatial and physical crowding during Hajj may attenuate this risk.

## Background

**Figure 1:**
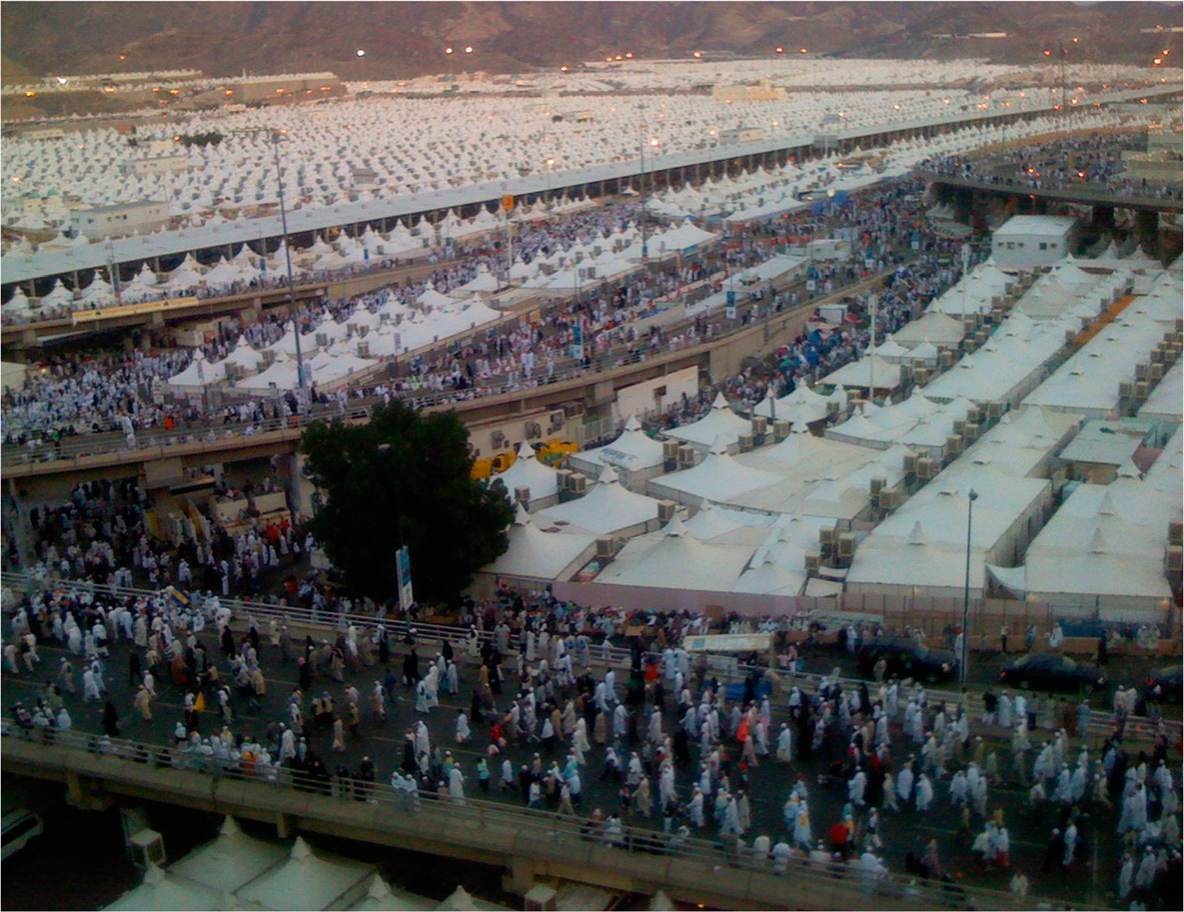
Tent housing facilities located in Mina inundated by pilgrims.

Hajj is the largest annual pilgrimage in the world, falling on the 12th lunar month of each year. The pilgrimage takes, on average, 7 days, but the period of Hajj is up to 21 days Pilgrims performing Hajj must perform specific rituals while following a specific route, i.e. performing the rituals of Hajj in a sequence other then the correct one will render one’s pilgrimage unsuccessful and incomplete, and in such cases the entire pilgrimage must be repeated. Pilgrims move from one holy site to another on–foot or sometimes by bus. Overcrowding, fatigue, extreme temperatures (ranging 24–37°C), intravascular volume and electrolyte disturbances contribute to illness during Hajj and may place pilgrims at increased risk for communicable diseases. One Hajj location, Mina, is approximately 2.9 square kilometers in size, and contains over 30,000 shared tent–type houses. These rectilinear structures ranged in size from 4x4m through to 8x12m. Each tent will usually be inhabited by 50 to 100 pilgrims (Figure 1). In comparison, the approximate areas of other set locations of housing include Makkah, at 850 (urban) to 1200 square kilometers (metro); Arafat, 10.4 square kilometers and Medina 589 square kilometers in area [1].

Pneumonia is a common and potentially life–threatening illness that continues to be a major global health problem, particularly among the elderly attempting Hajj [2]. Among infectious diseases, community acquired pneumonia (CAP) is the leading cause of death in the world and is associated with a substantial economic burden to healthcare systems [2]. Annually, approximately 151 million new cases of pneumonia are diagnosed worldwide and 11 million people require hospitalization. Treatment failure rates for pneumonia are as high as 15% [3-5]. Death rates from CAP increase with the presence of co–morbidities such as chronic heart failure, advanced chronic obstructive pulmonary disease (COPD), neurological diseases, cirrhosis, and advanced age [6-8].

Several studies have reported that respiratory tract infections are the leading types of infections during Hajj [9-11] and are a common cause of severe sepsis and septic shock necessitating ICU admission [12]. Pulmonary complications resulting from pneumonia are responsible for the second (after cardiovascular disease) greatest number of deaths during Hajj [9,10], resulting in a considerable burden on patients and the healthcare system. Furthermore, novel pneumonia acquired and spread during Hajj could lead to global outbreaks as over 2 million pilgrims return to their countries. In this study, we provide an account of pneumonia–related critical illness among hospitals providing medical care to Hajj pilgrims. We describe the number (and proportion) and temporal onset of severe pneumonia, patient characteristics, co–morbidities, severity of illness and clinical outcomes in addition to providing an overall incidence proportion for pneumonia-related critical illness during Hajj.

## Methods

### Study design & site

We prospectively designed a cohort study to occur over two consecutive Hajj years (November 18 to December 9 2009; November 7 to December 1 2010) and conducted on all patients admitted to ICU for at least 4 h. The primary outcome was the number (proportion of all ICU patients) and temporal onset for severe pneumonia. Secondary outcomes included patient co–morbidities, severity of illness and clinical outcomes, in addition to an overall incidence proportion (number of patients with pneumonia-related critical illness divided by the number of all Pilgrims) over the Hajj study period. Patients were followed from ICU admission until discharge, transfer or death, in the following centers: Al–Zahir Hospital, Makkah [39 beds], Heraa Hospital, Makkah [28 beds], King Faisal Hospital, Makkah [20 beds], Ajyad Hospital, Makkah [12 beds], Al–Noor Specialty Hospital, Makkah [24 beds], Al–Wadee Hospital, Mina [28 beds], Emergency Hospital, Mina [28 beds], Al–Jesser Hospital, Mina [28 beds], Al–Shareh Al–Jaded Hospital, Mina [18 beds], Arfat General Hospital, Arafat [28 beds], Nemrah Hospital, Arafat [12 beds], Al–Rahma Hospital, Arafat [18 beds], King Fahd General Hospital, Al–Medina [32 beds], Al–Ansar Hospital, Al–Medina [20 beds], and Ohoud Hospital, Al–Medina [12 beds]. The 15 centers participating in this study represent all fully functional and provisioned Saudi ministry of health hospitals servicing pilgrim during the study period. The hospitals in Mina and Arafat are temporary healthcare facilities that provide medical services only during the Hajj period while those in Makkah and Al–Medina are permanent facilities that provide healthcare services to patients throughout the year. The hospitals in Mina operate for a maximum of 2 weeks per year as opposed to the hospitals in Arafat that function for only 1 week per year; the duration of service depends mainly upon medical demand during Hajj.

### Study subjects and data collection

The acute physiology and chronic health evaluation (APACHE) score has been used worldwide for measuring ICU performance [13] and offers validated ICU risk–adjustment models for mortality and ICU length of stay [14,15]. Demographic, clinical, laboratory data, and variables necessary for the calculation of the APACHE IV score (vital signs, Glasgow Coma Scale, respiratory therapy variables, urine output, and laboratory data including serum glucose, sodium, arterial blood gas values, complete blood count, creatinine, urea, albumin and bilirubin), and procedures, including mechanical ventilation were collected by data collectors affiliated with the Hajj medicine operations of the Saudi Arabia Ministry of Health. The source of cultures for bacterial etiologic diagnosis of pneumonia was expectorated sputum and bronchoalveolar lavage when sputum was not available. Respiratory specimen analysis using real–time reverse–transcriptase–polymerase–chain–reaction (RT–PCR) assay was reviewed for patients with a clinical suspicion of influenza (including H1N1). Immunoassays were performed using the COBAS Influenza A test and the AMPLICOR Respiratory Specimen Preparation Kit (Roche, Inc). Patients were diagnosed with aspiration pneumonia at the discretion of an infectious diseases consultant and the attendant intensivist if they suffered the following: impaired swallowing; were on tracheal or nasogastric tube; impaired level of consciousness preceding the onset of pneumonia; changes seen on chest x-ray compatible with aspiration. The diagnosis of *Mycobacterium tuberculosis* was established in the present study both via clinical suspicion and later confirmed by acid-fast bacilli cultures. All Mycobacterium tuberculosis cases were confirmed via culture. Collected data were reviewed for completeness and accuracy by a committee of critical care physicians and respiratory therapists prior to database locking and analysis. The data was gathered on a previously piloted case report form, from ICU patient clinical files and laboratory results and then transcribed to a custom designed Microsoft Access database. APACHE–predicted and actual mortality rate and length of stay were determined for all patients. Calculation of the APACHE IV scores was done according to the predictor variables described by Zimmerman et al. [13] and with the aid of an online APACHE IV calculator, available on the Middle East Critical Care Assembly website [16]. We defined our short–term outcome as the three–week vital outcomes for all critically ill patients.

### Statistical analysis

This was primarily a descriptive study of the entire population of Hajj patients with critical illness and pneumonia; therefore, sample size calculations were not conducted *a priori*. The distribution of all variables was examined graphically and additionally with the Shapiro–Wilk test, to assess the normality of distribution for gathered variables. Categorical variables are presented as percentages and continuous variables as means (and standard deviations, SD) or medians (and interquartile range, IQR) as appropriate. Student’s t–test was used for comparisons of normal distributed continuous data and the Mann–Whitney *U* test and Kruskal–Wallis test were used for comparisons of non–parametrically distributed data. Actual and APACHE IV–predicted mortality was compared using both Student’s t–test and the Wilcoxon Rank Sum Score. For categorical variables, contingency tables were analyzed with the chi–square test, or Fisher’s exact test for small sample sizes. A *P*–value <0.05 was taken as statistically significant for individual variables. This study was approved by the research biomedical ethics board of the Saudi Ministry of Health.

## Results

### Baseline characteristics and temporality

**Figure 2:**
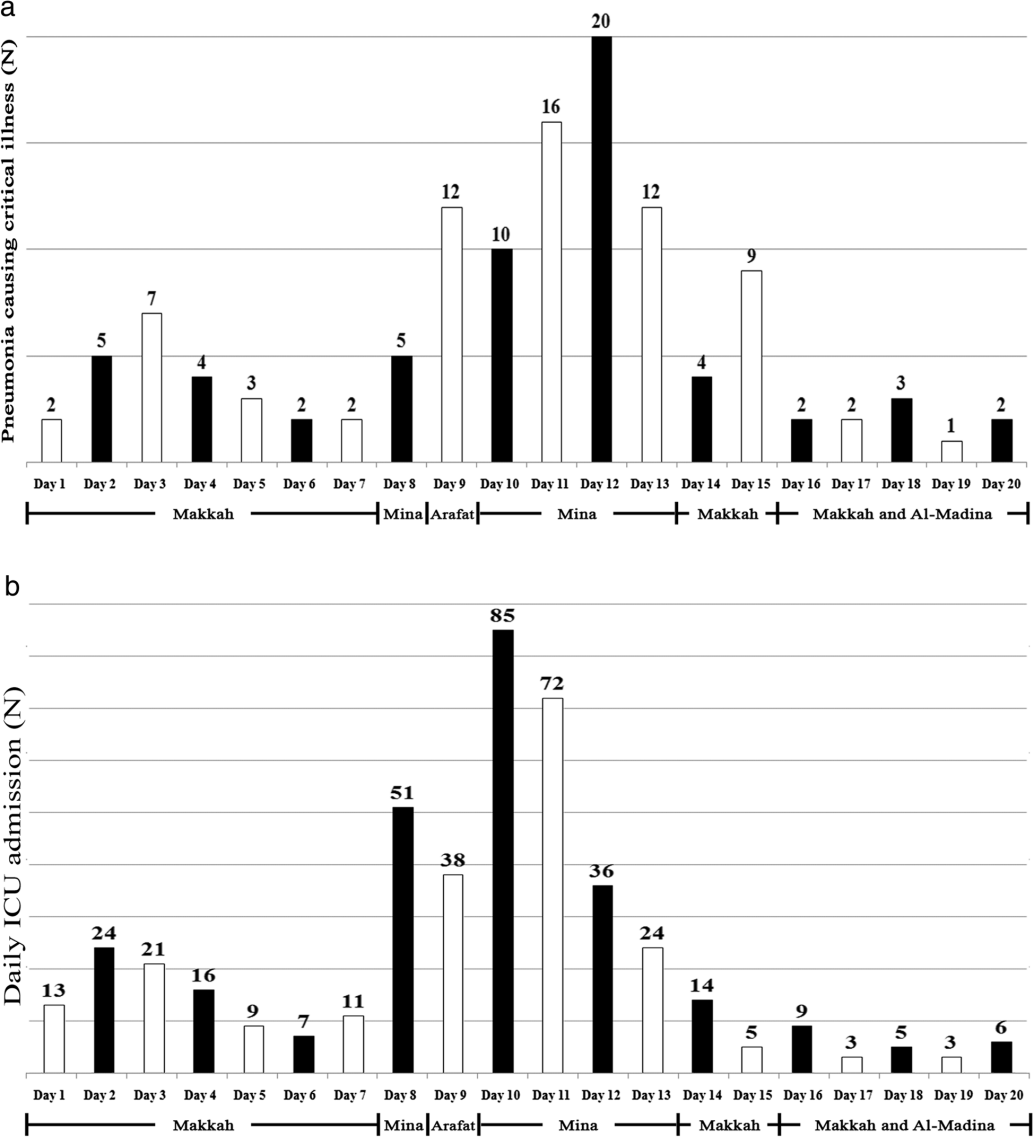
a. Pneumonia-related critical illness per day over the period of Hajj and the location of pilgrims during Hajj*. *Incident cases of pneumonia for each day represent the combined number of cases from both Hajj years. b. Combined number of ICU hospitalizations for each day of Hajj in two consecutive Hajj seasons.

The study population included 452 critically ill patients from over 40 nationalities. The mean (±SD) age was 64 ±12 years and 293 (64.6%) were male. There were 123 patients who developed pneumonia-related critical illness, accounting for 27.2% of all ICU admissions (Table 1), and an incidence proportion of 21.5 episodes of pneumonia-related critical illness per million pilgrims (123 episodes per 5.73 million over two years) during the 21-day period of Hajj. This corresponds to 62 cases of pneumonia-related critical illness per year or a mean of 2.9 cases of pneumonia-related critical illness per day of Hajj. Non-pneumonia causes of admission to ICU are listed in Appendix 1. Median APACHE IV score for critically ill patients with pneumonia at ICU admission was 81 (IQR 63–102). The etiological agents causing severe pneumonia in the present study were identified in 73 (59.4%) patients: gram–negative organisms were isolated in 22 (18%); gram–positive organisms in 13 (10.6%); fungus in 2 (1.6%); *Mycobacterium tuberculosis* in 6 (4.9%); and 30 (24.4%) had influenza A (H1N1), confirmed by PCR (Table 2). Among all cases of pneumonia, 88 (71.5%) occurred in the second week and 95 (77.2%) occurred between days 5–15 of Hajj (Figure 2a). Temporally, the vast majority of pneumonia occurred in the period following heaviest pilgrim density, among shared tenting, in Mina (Figure 2b).

**Table 1:** Demographic characteristics of 123 ICU pneumonia patients during Hajj, 2009 and 2010

**Characteristics**	**N (%) or Median (IQR)**
Age, years	64 ±12
**Age group**	
30 – 50	23 (18.7)
51 – 60	22 (17.9)
61 – 70	47 (38.2)
71 – 80	24 (19.5)
> 80	7 (5.7)
**Gender, male**	79 (64.2)
**Ethnicity**	
Black	28 (22.8)
East Asian	24 (19.5)
South Asian	42 (34.2)
Arab	26 (21.1)
White	3 (2.4)
**Type of Pneumonia**	
Hajj (Community) acquired	82 (66.7)
Aspiration	31 (25.2)
Nosocomial	4 (3.3)
Tuberculosis	6 (4.9)

Values are displayed as n (%), mean ± SD.

**Table 2:** Isolated bacteria among patients with pneumonia–related critical illness during Hajj, 2009 & 2010, and antibiotics sensitivity results

**Microorganisms isolated in sputum culture**	
*Microorganism*	*Number of positive cultures (n = 32)*
*Acinetobacter sp.*	8 (26.7%)
*Klebsiella sp.*	5 (16.7%)
*Pseudomonas aeruginosa*	5 (16.7%)
*Staphylococcus aureus*	3 (10%)
MRSA	3 (10%)
*Streptococcus sp.*	3 (10%)
*Escherichia coli*	3 (10%)
*Candida albicans*	2 (6.7%)
**Microorganisms isolated in blood cultures**	
*Microorganism*	*Number of positive cultures (n = 5)*
MRSA	4 (80%)
*Staphylococcus aureus*	1 (20%)
**Antibiotic sensitivity results**	
Sensitive to all antibiotics	3 (4.1%)
Resistance to one class of antibiotic	37 (50.7%)
Resistance to two classes of antibiotics	21 (28.8%)
Resistance to three or more classes of antibiotics	12 (16.4%)

Values are displayed as n (%).

**Abbreviations**: MRSA (methicillin–resistant *Staphylococcus aureus*).

### Concomitant illness

**Figure 3:**
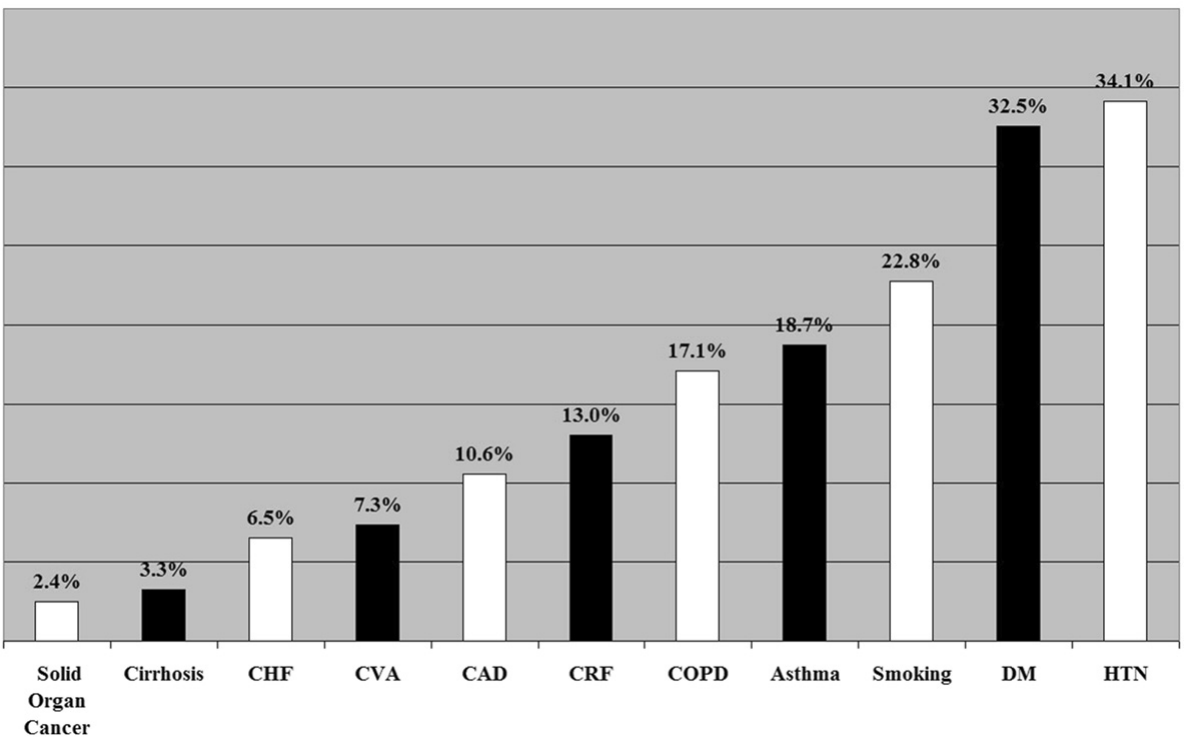
**Co–morbidities associated with Hajj ICU pneumonia patients. Abbreviations**: CHF (congestive heart failure); CVA (cerebrovascular accident); CAD (coronary artery disease); CRF (chronic renal failure); COPD (chronic obstructive pulmonary disease); DM (diabetes mellitus); HTN (hypertension).

Pneumonia was associated with considerable pre–existing co–morbidities (Figure 3) and was commonly concurrent with an exacerbation or onset of another medical problem – 51 patients (41.5%) had at least 2 active diagnoses, in addition to pneumonia. Twenty–one critically ill patients with pneumonia (17.1%) had active cardiovascular disease on ICU admission, 7 had atrial arrhythmias, 5 congestive heart failure, 4 ischemic changes on electrocardiograms, and 5 had symptomatic valvular heart disease identified via audible murmurs in the emergency triage areas and confirmed through echocardiograms performed in ICU. Twenty–six critically ill patients with pneumonia (21.1%) had new or exacerbated non–infectious pulmonary diseases on ICU admission: 10 with COPD exacerbations, 7 evolved to acute respiratory distress syndrome, and 9 had severe asthmatic attacks. Thirteen critically ill patients with pneumonia (10.6%) had neurological disorders on admission; 8 with transient ischemic attacks, 3 had metabolic encephalopathy, and 3 had severe peripheral neuropathy.

### Therapies received

A total of 85 patients (69.1%) received mechanical ventilation: 34 (40%) non–invasive ventilation; 31 (36.5%) assist–control ventilation; 12 (14.1%) controlled ventilation; 5 patients with invasive continuous positive airway pressure; and 3 were treated with high frequency oscillatory ventilation. Median duration of mechanical ventilation was 4 (IQR 3–6) days, with a maximum of 13 days.

### Clinical outcomes

Short–term (3-week) mortality was 16.2% among all critically ill patients during Hajj, and 19.5% among all patients with pneumonia (*P* = 0.34). Median APACHE IV *predicted* mortality for all patients was 18% (95% confidence interval 24.6–29.1%) and 29.6% (95% confidence interval 28.8–37.6%) for patients with pneumonia. Actual (short–term) mortality was significantly less than predicted longer–term mortality ( *P* = 0.007). Median APACHE IV score on ICU admission for critically ill patients with pneumonia was 78 (IQR 58.5–96) for patients who eventually survived and 102 (IQR 79.5–123.5) among patients who died ( *P* < 0.0001). Actual mortality was highest among multidrug resistant isolated organisms (4/12, 33%), compared to non-resistant (0), those resistant to one type of antibiotics (10/37, 27%), and those resistant to two types of antibiotics (6/21, 28.6%) ( *P* = 0.71). Overall, of 123 patients with pneumonia, 84 (68.3%) were discharged from the ICU to the hospital ward without residual complications, 27 (22%) were referred to specialty centers for advanced care, and 12 (9.6%) were discharged home, 3 of whom against medical advice (Table 3). Length of stay in ICU at Hajj centers was 4 (IQR 1–8) days in comparison to APACHE IV predicted length of stay of 6.3 (IQR 5.3–7.3) days (*p* = 0.007); many of these patients were transported outside of Hajj temporary facilities during the pilgrimage.

**Table 3:** Outcomes of Patients with pneumonia–related critical illness during Hajj, 2009 & 2010

**Outcome**	**N (%) or Median (IQR)**
Actual mortality (at 3 weeks)	24 (19.5%)
APACHE IV predicted mortality	36 (29.6%, IQR 11.1%–49.4%)
**Primary Cause of Death**	
*Primarily Infectious*	8 (33.3)
Septic shock	6 (25)
Lung abscess	2 (8.3)
*Pulmonary, secondary to pneumonia*	14 (58.3)
Acute respiratory distress syndrome	11 (45.8)
Hypoxia or severely impaired ventilation	3 (12.5)
*Cardiac arrhythmia*	2 (8.3)

Values are displayed as n (%), median (IQR).

## Discussion

In this large cohort study of all pilgrims during two consecutive Hajj seasons, we found that among 452 critically ill patients, pneumonia was the reason for admission to ICU in 27%. Importantly, the vast majority of pneumonia–related critical illness is acquired *during* Hajj and temporally relates to extreme spatial crowding during site–to–site migration and shared nighttime tenting [17,18]. This represents a substantial burden of illness for travelers, Saudi Arabia healthcare facilities, and potentially for the healthcare jurisdictions to which pilgrims will return.

From a public health perspective, Hajj could facilitate dissemination of influenza or other infections around the globe [19]. It has been estimated that more than one in three pilgrims will experience respiratory symptoms during their stay in Mina [20]. Recent work highlighting the high rates of infection and carriage of influenza virus in pilgrims returning from Mecca emphasizes the need for international cooperation and strategies to minimize this risk [20-22]. Hajj accommodation is, of necessity, in tents in the desert plains of Mina, and it is not unusual for 50–100 people to share a single tent at night [23]. Such overcrowding and continuous close contact greatly increases the spread of respiratory infections.

Pneumonia prevention should be an increased focus of Hajj medicine. Vaccination against seasonal and pandemic influenza strains in addition to Pneumococcal vaccine should be considered for at–risk patients before Hajj. Intra–pilgrimage frequent hand washing, and potentially the wearing of face masks for patients with early symptoms, should be a future focus of Hajj medicine in order to reduce the burden of respiratory tract infection. Also, improvements in pilgrim living conditions with less spatial crowding and decreased density of pilgrims in shelters in Mina may decrease the incidence of transmission of severe pneumonia in Hajj and should be explored further. Beyond the period of Hajj, patients who have returned from the pilgrimage (and clinicians evaluating them) should be vigilant for signs of pneumonia.

Knowledge about the etiological agent of pneumonia–related critical illness is an important but challenging step in the management of pneumonia in such mass gatherings [9-11]. Median length of stay for ICU pneumonia patients in our cohort was 4 (IQR 1–8) days. During this period it was practically difficult to isolate organisms from all patients due to early initiation of antimicrobial therapy prior to sampling, and limited capacity for testing in temporary hospital laboratories. The massive influx of patients on hospitals generally and ICUs specifically posed significant challenges to ICU physicians and nurses collecting more detailed data on critically ill patients. Several studies have reported on the occurrence of pneumonia and its etiological agents in *noncritically ill patients* during Hajj. Ashgar et al., reported that *Candida albicans* (28.7%), *Pseudomonas aeruginosa* (21.8%), and *Legionella sp.* (14.9%) were the most frequent isolates in three hospitals during Hajj and that *Mycobacterium tuberculosis* accounted for 1% of pneumonia cases [24]. In our cohort, prevalence of tuberculosis was similar at 4.9%. Madani et al., reported that pneumonia accounted for 22% of all ICU admissions to Mina and Arafat hospitals during Hajj and that tuberculosis accounted for 5.9% of pneumonia cases [9]. Alzeer et al., reported that tuberculosis (20%), Gram–negative organisms (18.8%) and *Streptococcus pneumonia* (10%) were the frequent isolates from patients admitted to two hospitals in Makkah during Hajj, and concluded that the etiological agents of pneumonia during Hajj are different from those of more typical community acquired illness [11]. We recommend that future studies focus on identifying the microbiological etiology of critical illness–related pneumonia in Hajj, which might help estimate the impact of specific prevention and also guide empiric therapy during Hajj.

Pilgrims may be at higher risk of acquiring very transmissible organisms, such as respiratory viruses, certain bacteria, and *Mycobacterium tuberculosis,* during Hajj. This has significant public health implications for tuberculosis control in countries with large Muslim populations. The intense congestion, living in close proximity with vast crowds and the increasing percentage of elderly pilgrims, are all factors potentially magnifying this risk. Additionally, many Muslims travel from countries where tuberculosis is highly endemic. A recent study conducted on Malaysian pilgrims revealed that 10% displayed a significant increase in immune response to QuantiFERON tuberculosis assay antigen post–Hajj when compared to pre–Hajj [25].

Over the past decade, several risk factors for pneumonia–related morbidity and mortality have been detected [26,27]. A number of studies reported that occurrence of pneumonia in diabetic patients was associated with a poor prognosis [28,29]. Our study confirms that older ages, diabetes, smoking, chronic conditions such as asthma and COPD and an immune compromised state are conditions commonly associated with pneumonia–related critical illness. The vast majority of these patients required mechanical ventilation and some suffered severe oxygenation failure. Despite high severity of illness by APACHE IV scores, short–term mortality during the period of Hajj was favorable to that predicted, highlighting that with sufficient planning and provision of advanced temporary critical care services, severe pneumonia and respiratory failure can be managed under challenging circumstances such as mass gatherings. However, it is important to note that the ultimate mortality rate among critically ill patients in our cohort is likely to be underestimated by the ICU mortality as some patients may have died after transfer from ICU to the ward or to other hospitals.

## Conclusion

Pneumonia is a major cause of critical illness during Hajj and occurs amidst substantial crowding and pilgrim density. Increased efforts at prevention for at–risk patients prior to Hajj and further attention to spatial and physical crowding during Hajj may attenuate this risk. We also found that severe pneumonia during Hajj is associated with considerable co–morbidities and these may prove helpful in identifying patients at increased risk prior to travel to Saudi Arabia. Future studies should focus more heavily upon prevention, in addition to diagnosis, etiology, and management of severe pneumonia during Hajj.

## Appendix

### Appendix 1. Non-pneumonia causes of admission to ICU among 452 critically ill patients

*Some patients had more than one admitting diagnosis; hence, the total number is greater than 452 patients.

## Abbreviation

DM, Diabetes mellitus; COPD, Chronic obstructive pulmonary disease; ICU, Intensive care unit; CAP, Community–acquired pneumonia; APACHE, Acute physiology and chronic health evaluation; AP, Aspiration pneumonia; IQR, Interquartile range.

## Competing interests

The authors declare that they have no competing interests.

## Author contributions

All authors, external and internal, had full access to all of the data (including statistical reports and tables) in the study and can take responsibility for the integrity of the data and the accuracy of the data analysis. YM, AO, and SO contributed to data collection. AA, AO, SO, and RF contributed to the design of the study. AO and RF contributed to the data analysis and data interpretation. YM, AA, AO, SO, and RF participated in the writing of all sections of the manuscript. All the authors read and approved the final version of the manuscript, including all required revisions.

## Pre-publication history

The pre-publication history for this paper can be accessed here:

http://www.biomedcentral.com/1471-2334/12/117/prepub
